# Matrimid Mixed Matrix Hollow Fiber Membranes: Influence of ZIF‑8 Filler over O_2_/N_2_ Separation Performance

**DOI:** 10.1021/acsomega.4c11696

**Published:** 2025-05-29

**Authors:** Daniel González-Revuelta, Marcos Fallanza, Alfredo Ortiz, Daniel Gorri

**Affiliations:** Departamento de Ingenierías Química y Biomolecular, 16761 Universidad de Cantabria, Av. de los Castros s/n, Santander 39005, Spain

## Abstract

Oxygen and nitrogen are two valuable and widely used products in industries, medical applications, and the food sector, for instance, so they present a significant market opportunity. Traditional methods to separate these gases are very energy-intensive. Therefore, in a world moving toward energy sustainability, membrane technology offers a more sustainable alternative. The aim of this work is to develop polymeric membranes with a hollow fiber configuration using Matrimid and ZIF-8 as fillers to improve the separation performance. As a first step, planar membranes with varying ZIF-8 content (0–20 wt %) were prepared using the casting solvent evaporation technique to determine the optimal Matrimid/ZIF-8 ratio. Subsequently, hollow fiber membranes of Matrimid and Matrimid/ZIF-8 were fabricated via the spinning method. The novelty of this work lies in incorporating ZIF-8 into Matrimid membranes in a hollow fiber configuration, as it is known that the inclusion of fillers in polymeric membranes allows improvements in their performance. Nevertheless, their production is quite challenging. The flat-sheet membranes prepared have shown that inclusion of ZIF-8 in Matrimid improves its O_2_/N_2_ selectivity by one point and that 5 wt % loading is the optimal point to see this enhancement. The same effect was observed in hollow fiber membranes, reaching an oxygen permeance of 2.16 GPU and an O_2_/N_2_ selectivity of around 6 (30 °C) for the Matrimid/ZIF-8 membrane, with a dense layer thickness of around 2 μm. All of these results were compared with those reported in the literature for the O_2_/N_2_ separation. Additionally, the impact of feed composition variations on membrane performance was analyzed.

## Graphical abstract

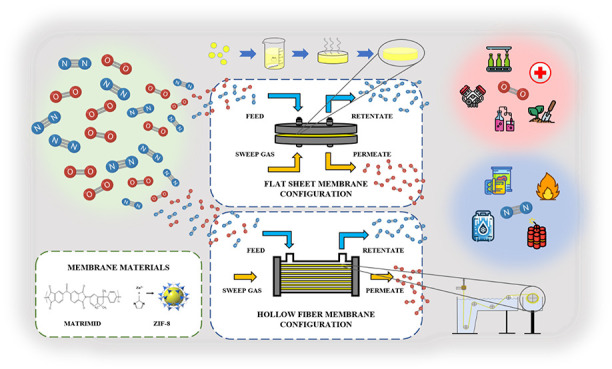

## 1 Introduction

The production of oxygen and nitrogen has grown in recent years due to their wide use in different industries, which has led to continuous improvement in the production techniques of both compounds. Their versatility makes them a popular choice for a broad range of industrial applications. On one hand, oxygen is extensively used within the iron and steel industry and the chemical sector as a reagent, and it also finds significant application in the field of medicine.^1,2^ In addition, pure oxygen plays a crucial role in oxy-fuel combustion, where oxygen is employed as a substitute for air in the fuel combustion, leading to zero carbon emissions.^3^ From another standpoint, the principal attribute that renders nitrogen an attractive choice lies in its low reactivity with other compounds, thereby making it inert. Consequently, it finds widespread utility in the food industry, where it serves as an inert atmosphere, and also in cryogenics and medical care applications.^4^

The global industrial oxygen market is currently valued at US$ 70 billion, and it is projected to double in the next 10 years. This means that industrial oxygen is one of the world́s most in-demand gases, and for the foreseeable future, this will increase considerably and its production will constitute a major market opportunity.^5^ For both oxygen and nitrogen, atmospheric air is the primary source. Depending on the required production volumes and purity levels for each application, different separation methods are employed. Traditionally, the most prevalent separation techniques include cryogenic distillation and pressure swing adsorption. The former has already reached a very high level of maturity and is the most widely used on an industrial scale, as it allows high oxygen purities (>99%), but this technology is known for being complex, expensive, and highly energy-intensive. Adsorption also achieves high purities (up to 95%); however, the requirement for adsorbents limits its capacity mainly due to capital costs.^6–8^

Both of these techniques have little room for improvement in the future, and in a world moving toward energy sustainability, new methods need to be developed to improve the production efficiencies of these compounds. In this regard, membrane technology holds great promise for separating oxygen and nitrogen from air.^9^ This technique offers several advantages over the two previous ones, including the possibility of operating at different scales, reduced energy requirements, and lower production costs.^10–12^ Nevertheless, the greatest challenge faced by this technology is the separation of two molecules with such similar kinetic diameters as oxygen and nitrogen, 3.46 Å and 3.64 Å, respectively.^13^ Membranes used for gas permeation must have favorable inherent transport properties and mechanical strength under adverse thermal and feed mixture conditions. Moreover, it is also crucial to understand the relationship between the properties of each polymer and transport of the target gas through it. There are several types of membranes depending on the materials used for their manufacture, but the most studied and the ones in current commercial use are dense polymeric membranes.^14^ Among all of the existing polymeric materials, two types can be differentiated: glassy and rubbery polymers. The main difference is that the former usually exhibit greater selectivity due to their rigidity and very compact polymer chains, while the latter gain permeability at the expense of losing selectivity because they have a more flexible and elastic structure at room temperature. The polymers that exhibit good performance in the separation of oxygen and nitrogen and are widely studied in the literature include polysulfones (PSs), poly­(ether sulfones) (PESs), polyimides (PIs), and poly­(ether imide)­s (PEIs), among others. Polysulfone has an oxygen permeability of 1.4 Barrer and an O_2_/N_2_ selectivity of 5.6 (at 35 °C),^15^ while a polyimide such as 6FDA-ODA presents an oxygen permeability of 4.3 Barrer, with an O_2_/N_2_ selectivity of 5.2 (at 35 °C).^16^ Matrimid is another polyimide that exhibits excellent separation performance, with an oxygen permeability of 1.8 Barrer and O_2_/N_2_ selectivity of around 6.^17^ This material presents a highly promising permeability–selectivity relationship compared with other materials, making it a strong candidate for potential industrial implementation. As a result, this study focuses on its development for membrane production.^18^

But those materials, on their own, do not present sufficient performance in terms of permeability and selectivity to be competitive at an industrial level.^19^ That is why, in recent years, research has focused on composite mixed matrix membranes (MMMs), which consist of a polymeric part combined with fillers, such as molecular sieve materials. This type of membrane combines the cost-effectiveness of polymeric materials with the high separation performance of molecular sieve materials.^20,21^ The inclusion of fillers in polymeric flat-sheet membranes for gas separation has been extensively studied over the past few years, confirming the improved performance of membranes when these materials are included. Among the candidates, zeolites and molecular carbon sieves are the most commonly used inorganic fillers in MMMs because they are able to help the polymer effectively separate gas molecules with similar sizes.^22,23^ Li et al.^24^ included zeolites with different loadings and sizes as fillers in a poly­(ether sulfone) (PES) polymeric matrix for O_2_ and N_2_ separation, finding a decrease in gas permeability with increasing filler loading. In another work, Yong et al.^25^ studied the inclusion of zeolites in Matrimid for O_2_/N_2_ separation and achieved a significant increase in O_2_ permeability (from 1.5 to 6.6 Barrer), but at the cost of a loss in selectivity from 6.9 to 4.9. Other authors have investigated the inclusion of zeolitic imidazolate frameworks (ZIFs), which are comprised of a subset of MOFs and exhibit exceptional thermal and chemical stability.^26–28^ Song et al.^29^ studied the improvement in performance for gas permeation on flat-sheet Matrimid membranes by incorporating ZIF-8 and modifying the annealing temperatures. As a conclusion, they observed that the best annealing temperature is 230 °C and that the addition of this filler significantly improves the membrane behavior.

However, with the final aim of making membrane technology competitive at the industrial level, significant efforts are being made to move from a planar configuration to a much more favorable one: the hollow fiber configuration. The primary reason is that this configuration allows for higher packing density and greater ease of module fabrication compared to the flat-sheet configuration.^30,31^ The main challenge in producing hollow fiber membranes is the creation of defects on their surface, so in some cases, it is necessary to use spin dopes with high concentration and viscosity during the spinning process to reduce their possible formation.^32^ Chen et al.^18^ investigated the permeation performance of various gases for several polymers in the hollow fiber configuration, including Matrimid. In developing their hollow fiber membranes, they had to seal the defects on the outer surface with a cover layer of silicone rubber (PDMS). Additionally, the inclusion of fillers to prepare mixed matrix membranes in the hollow fiber configuration is an area that has been emphasized in recent years due to its promising performance. As previously mentioned, ZIF-8 is one of the best fillers in mixed matrix membranes. However, there are not yet many studies that include it in hollow fiber membrane configurations. Dai et al.^33^ conducted a study in which they included ZIF-8 as an additive to poly­(ether imide) (Ultem 1000) membranes in the hollow fiber configuration for CO_2_ and N_2_ separation. They achieved this by coextruding an inner polymer layer and an outer layer containing 4.5% wt. ZIF-8 in the spin dope. In a recent article, the authors reported the preparation of hollow fibers based on Matrimid with the addition of ZIF-8 as a filler.^34^ In that work, the membrane separation performance was tested for the selective separation of hydrogen, and it was found that the addition of 5% ZIF-8 to the polymer matrix led to a significant improvement in permeances, while the H_2_/CO_2_ selectivity increased by around 40%. The separation of the O_2_/N_2_ mixtures can be considered more challenging than the selective separation of hydrogen from its gaseous mixtures. Regarding the separation of O_2_/N_2_ mixtures, although some contradictory results have been published in the literature, the authors have selected ZIF-8 as a filler taking into account some experimental studies whose results support the idea about the positive effect of adding ZIF-8 into the polymeric matrix to improve O_2_/N_2_ selectivity.^26,35,36^ In a recent study, Hadi et al.^26^ included ZIF-8 as a filler in different proportions in poly­(ether sulfone) hollow fiber membranes for O_2_/N_2_ separation. The results showed that an increase in ZIF-8 concentration up to 10% increased O_2_ permeability compared to that of N_2_, leading to a considerable increase in selectivity (from 2.5 to 5). As the ZIF-8 concentration increased above 10%, the selectivity decreased considerably due to its agglomeration and the creation of defects in the selective layer.

As observed, Matrimid is a material that exhibits excellent performance in the separation of oxygen and nitrogen, which is why it has been selected as the base material for the membrane developing in this work. Similarly, ZIF-8 is a highly promising additive to be included in different polymeric materials for the separation of these two compounds. Based on this premise, this study focuses on the development of Matrimid-based mixed matrix membranes, including ZIF-8 as a filler. To achieve this, membranes were first developed in a flat-sheet configuration using pure Matrimid and different proportions of ZIF-8, in order to study the effect of varying its concentration. Subsequently, pure Matrimid and Matrimid/ZIF-8 membranes were produced in a hollow fiber configuration by using the spinning method. In addition to reporting the technique for the preparation of defect-free Matrimid hollow fiber membranes and the inclusion of ZIF-8 as an additive in Matrimid hollow fiber membranes, the novelty of this work lies in the fact that specific results are reported for the separation of O_2_/N_2_ mixtures obtained from real gaseous mixtures, investigating the optimal concentration of ZIF-8 in Matrimid membranes and showing the influence of several operational variables such as temperature, pressure, and mixture composition on the separation performance.

## 2 Results

### 2.1 Morphology

The morphology of ZIF-8 used has been studied in addition to that of membranes developed in this work, in both flat-sheet and hollow fiber configurations.

#### 2.1.1 ZIF-8 Morphology and Structure

In order to characterize the material used as a filler in this work, ZIF-8, the following analytical techniques were used. First, scanning electron microscopy (SEM) was employed to observe its structure. Additionally, thermogravimetric analysis (TGA) was used to study its thermal resistance and stability. Figure 1 shows the particles of ZIF-8 at different scales. It can be seen how the ZIF-8 particles tend to form a network of polycrystalline structures. In addition, small debris or impurities can be observed around these crystals.

**Figure 1:**
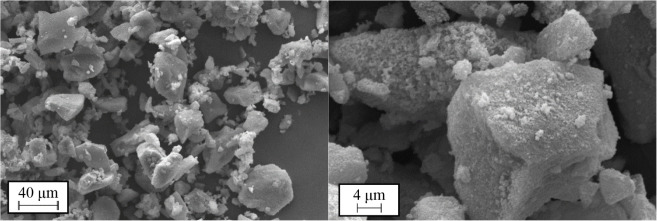
SEM images of the ZIF-8 samples.

As for the thermogravimetric analysis, the stability and thermal resistance of ZIF-8 were studied under two different environments: an inert atmosphere based on nitrogen and an oxidative atmosphere such as air, which are close to the conditions to be studied in this paper (mixtures of oxygen and nitrogen). The temperature ramp used was 5 °C/min. Figure 2 shows the TGA results for the ZIF-8 sample in both environments. As can be seen, a small weight loss of about 5% is observed as the temperature increases up to 200 °C, which corresponds to the presence of residues or traces of moisture. It can also be seen that ZIF-8 is stable up to a temperature of almost 400 °C under an air atmosphere and up to about 500 °C in an inert environment. Beyond these temperatures, the structure is lost until about 30% of the weight remains at 500 °C in air and 800 °C in nitrogen. These results are in agreement with those reported in the literature.^37,38^ From these results, it can be concluded that ZIF-8 is more stable under inert atmospheres. Nevertheless, the thermal resistance shown by this material does not compromise the application used in this work.

**Figure 2:**
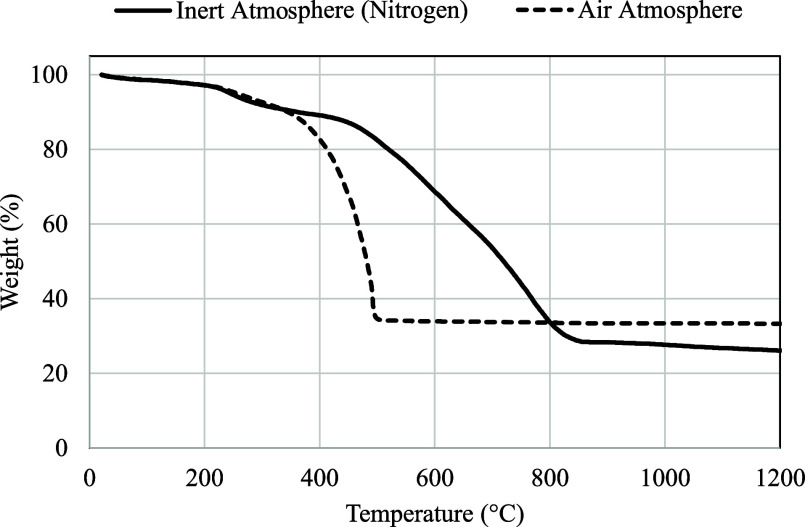
TGA results for the ZIF-8 samples under nitrogen and air atmospheres.

#### 2.1.2 Flat-Sheet Membrane Morphology

In this work, five different types of flat-sheet membranes have been prepared. First, a membrane composed solely of Matrimid, which serves as the base material. Additionally, different filler loads (ZIF-8) have been incorporated at weight ratios of 5%, 10%, 15%, and 20%. The morphological characterization of all of these membranes was conducted using scanning electron microscopy (SEM). In Figure 3, a comparison of SEM images is presented for the pristine Matrimid membrane and for the one with 5 wt % of ZIF-8. These images include both surface views (Figure 3a,c) and cross-sectional views (Figure 3b,d). Additionally, Figure 3e,f shows a zoomed-in view of the cross section and its EDX analysis of the Matrimid + 5 wt % ZIF-8 membrane, corresponding to a mapping performed to check the spatial distribution along the section of the zinc element, which is one of those included in the ZIF-8 filler. This is done in order to see if there are any particle agglomeration or voids between the particle and the polymer.

**Figure 3:**
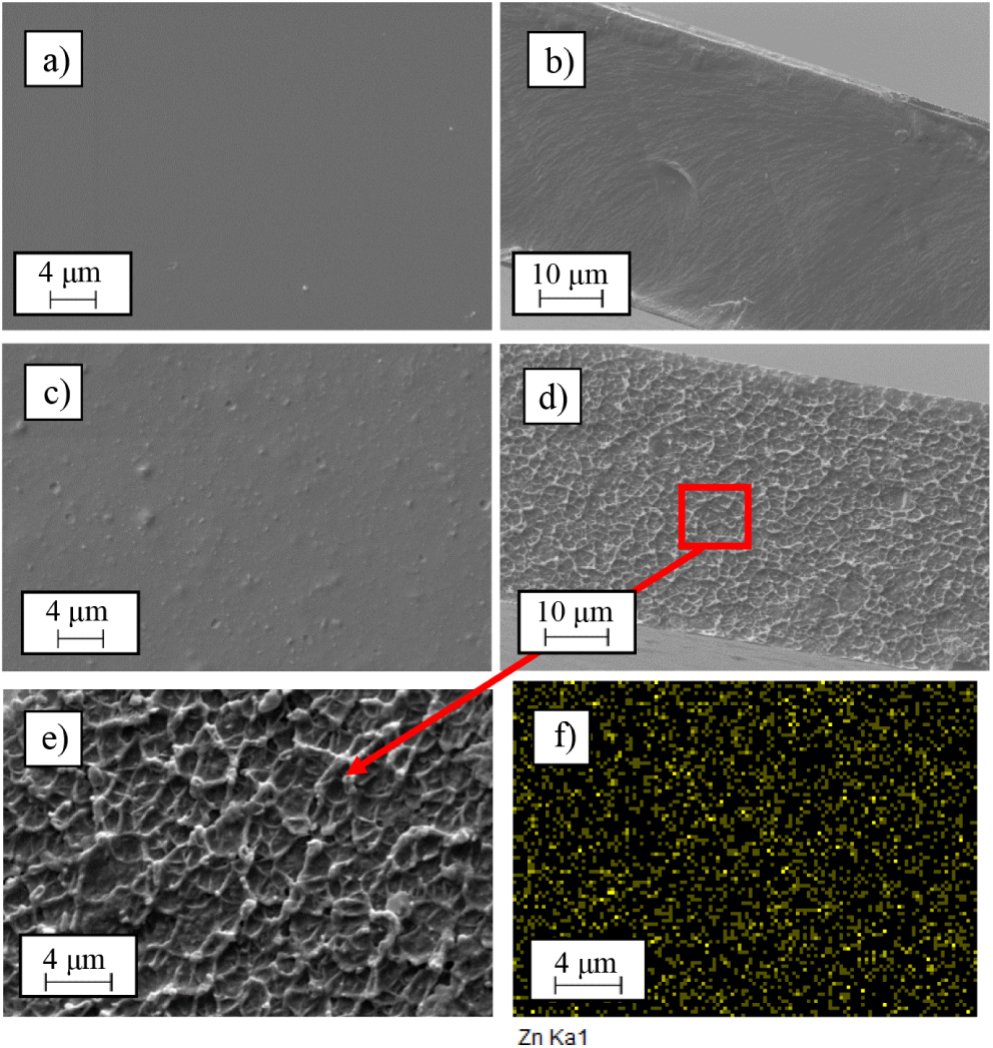
SEM images: external surface (a) and cross section (b) of the Matrimid membrane and external surface (c), cross section (d), zoomed-in view of the cross section (e), and zinc EDX analysis of the zoomed-in view of the cross section (f) of the Matrimid + 5 wt % ZIF-8 membrane.

As observed, the developed membranes are completely dense and exhibit no apparent defects. The small dots visible in Figure 3c result from the addition of ZIF-8, but they do not adversely affect the membrane performance. Regarding the thickness of the flat-sheet membranes used in this study, they are all around 50 μm, as confirmed by the cross-sectional images (Figure 3b,d). Furthermore, these images reveal how Matrimid undergoes a structural change when ZIF-8 is added. While the base structure of Matrimid (Figure 3b) has a soft and smooth appearance, the addition of ZIF-8 (Figure 3d) creates small tunnels, resulting in a significantly different structural network distributed homogeneously throughout the membrane. In view of Figure 3e,f, it can be affirmed that Matrimid and ZIF-8 are compatible materials as ZIF-8 is uniformly distributed across the entire membrane without observable agglomerations, which could otherwise hinder membrane separation performance. This observation holds true across the weight ratios studied, from 5 wt % to 20 wt % ZIF-8.

Finally, in the EDX analysis of the cross-sectional membrane with fillers, zinc is perfectly distributed throughout the entire membrane. This confirms the visual perception that ZIF-8 is well-dispersed without any agglomerations.

#### 2.1.3 Hollow Fiber Membrane Morphology

The transition from flat-sheet membranes to a hollow fiber configuration is poised to significantly enhance their performance, which could position membranes as an industrially competitive technology for gas separation. For this reason, two types of membranes in a hollow fiber configuration have been developed in this work: on one hand, a hollow fiber membrane composed solely of Matrimid and, on the other hand, a membrane consisting of Matrimid with a 5 wt % filler loading of ZIF-8. Unlike planar membranes, with the hollow fiber membranes, it has been decided to work only with 5 wt % filler content and not to increase it because, as will be seen in the Results section, this load is sufficient to see notable improvements in membrane performance, and increasing it does not lead to improvements in permeability and selectivity. Additionally, considering the economic viability of membrane production, the inclusion of low loading of ZIF-8 is beneficial as it is a relatively high-cost material. Figure 4 shows SEM images of both full and partial cross sections of the two hollow fiber membrane types, along with a zoomed-in view of the dense layer area and its zinc EDX analysis.

**Figure 4:**
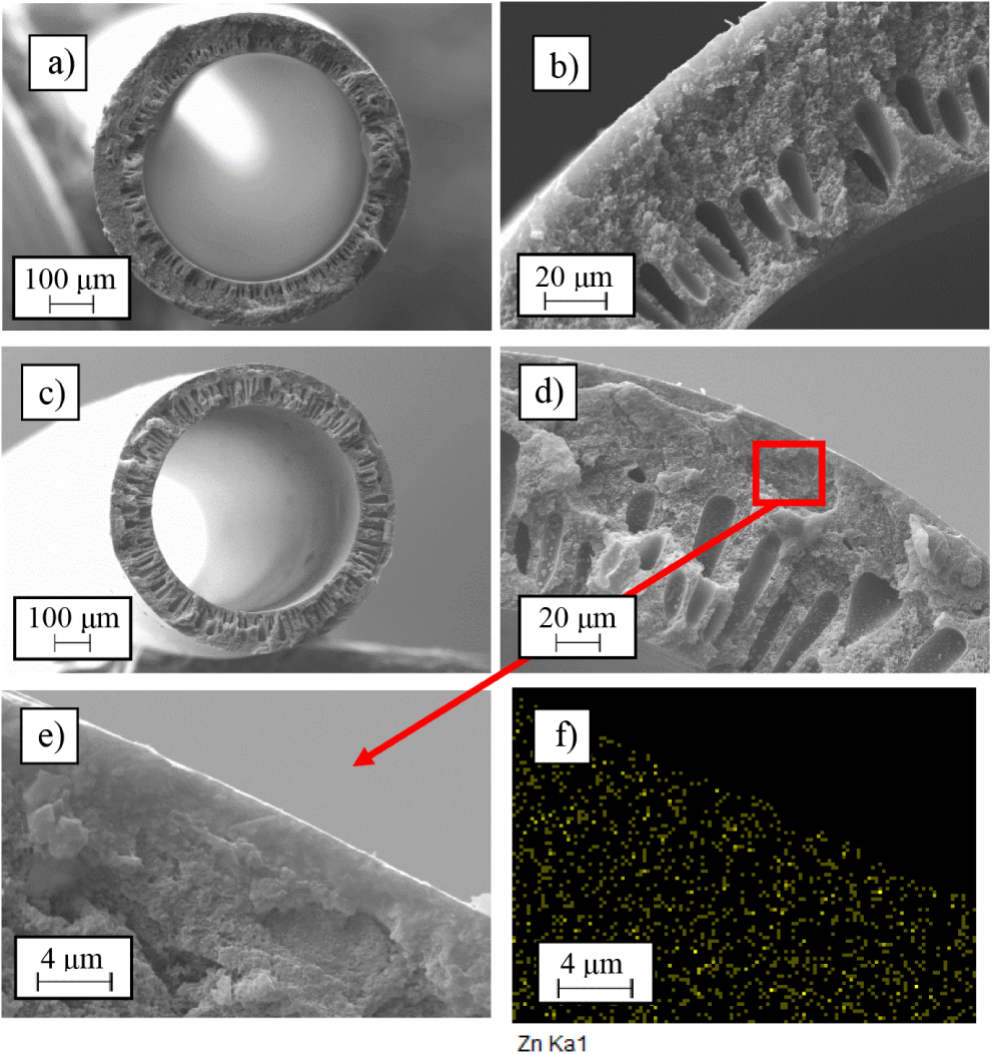
SEM images: general cross section (a) and cross-sectional detail (b) of the Matrimid hollow fiber membrane and general cross section (c), cross-sectional detail (d), zoomed-in view of the dense layer (e), and zinc EDX analysis of the zoomed-in view of the dense layer (f) of the Matrimid + 5 wt % ZIF-8 hollow fiber membrane.

The spinning conditions used for both membranes were the same, as already mentioned in the Methodology section, with the aim of evaluating the improvement in the membrane performance by adding ZIF-8. However, the cross-sectional sizes obtained for both are different. While the pristine Matrimid membrane has an outer diameter of about 730 μm with a wall thickness of 100 μm, the Matrimid/ZIF-8 membrane has an outer diameter of 900 μm and a wall thickness of 175 μm, which are considerably larger. This effect is due to the higher viscosity of the Matrimid/ZIF-8 solution when spinning. As shown in Figure 4, the morphology is similar for both membranes, with a highly porous substrate that provides structure and good mechanical strength to the membrane without hindering separation, which is handled by a thin selective dense layer on the outer surface. The macroholes present in the internal part of the membrane are produced by the action of the bore liquid used, while in the external part, the dense selective layer is produced by the use of water as an external coagulant, as well as by the use of a high air gap in the spinning process. This dense layer can be seen in Figure 4b,d for both membranes. Although the thickness of this layer cannot be measured accurately, a dense zone on the outer section of a few micrometers can be inferred, achieving the goal of developing a sufficiently thin layer without defects. It is important to note that the spinning process should be carried out while maintaining the polymer solution temperature constant at around 50 °C. If the temperature drops significantly, it would affect the viscosity of the polymer solution and thus hinder the whole process, while if the temperature increases too much, it would produce defects in the hollow fiber membranes.

Regarding the Matrimid/ZIF-8 hollow fiber membrane, Figure 4d clearly shows small dots throughout the hollow fiber body, corresponding to the filler. In addition, Figure 4e shows a zoomed-in view of the outer area of the membrane, where the dense selective layer is located and can be seen in more detail. To confirm the correct distribution of ZIF-8 along the membrane and in order to verify its presence in the dense layer, Figure 4f shows the EDX analysis of the cross section corresponding to the vicinity of the dense layer. The yellow dots correspond to the presence of zinc, of which ZIF-8 is composed, and as can be seen, there are no agglomerations, indicating that ZIF-8 is well distributed throughout the membrane body and the dense layer. Thus, as with flat-sheet membranes, good compatibility between Matrimid and ZIF-8 is also observed in the hollow fiber configuration. It is well-known that when trying to make asymmetric hollow fiber membranes with a dense layer of small thickness, the formation of microdefects is frequent, as reported in a previous work by the authors^9^ and in different works in the literature.^18,39^ In this case and in view of the data to be presented in the Results section, the developed hollow fiber membranes do not present defects in the selective layer.

### 2.2 Performance

Membrane performance is evaluated in terms of permeability, permeance, and oxygen/nitrogen selectivity.

#### 2.2.1 Flat-Sheet Membrane Performance

In this case, permeability can be determined thanks to the fact that the thickness of the selective dense layer can be measured, while permeance allows for a comparison of productivity between these and hollow fiber membranes. The study temperatures ranged from 30 to 70 °C, while the partial pressure gradient of each compound was up to 3.5 bar. In order to evaluate the performance of the membrane in terms of permeability, and according to eq 2, the relationship between permeate flux and the partial pressure gradient of each compound allows for the determination of permeance for each of them and for each temperature. As an example, Figure S1 shows the O_2_ flux vs the partial pressure gradient of O_2_. A linear relationship between flux and driving force can be observed for each temperature, implying that the permeance is independent of the pressure in the working range. Similarly, this has been obtained for N_2_ and for the other membranes. By obtaining the permeance and knowing the thickness of each membrane, we can obtain permeability according to the relationship in eq 4. As can be seen, the dependence of oxygen flux through the membrane is considerably influenced by temperature, with the flux increasing as the temperature rises.

As previously mentioned, five types of polymeric membranes in the flat-sheet configuration have been developed in this work: one with a matrix consisting solely of Matrimid and four others consisting of Matrimid with different ZIF-8 loadings (5, 10, 15, and 20 wt %). The ultimate goal is to compare the improvement in terms of oxygen permeability and O_2_/N_2_ selectivity that the material acquires by adding ZIF-8 and how the membrane behavior changes with the variation of this loading. Figure 5 shows a comparison between the different flat-sheet membranes in terms of the O_2_ permeance and the O_2_/N_2_ selectivity for different temperatures when working with a gas mixture feed of oxygen and nitrogen with a 50:50 mol ratio. The numerical values represented in this figure are provided in Table S1, together with the O_2_ permeance and the thickness of the membranes.

**Figure 5:**
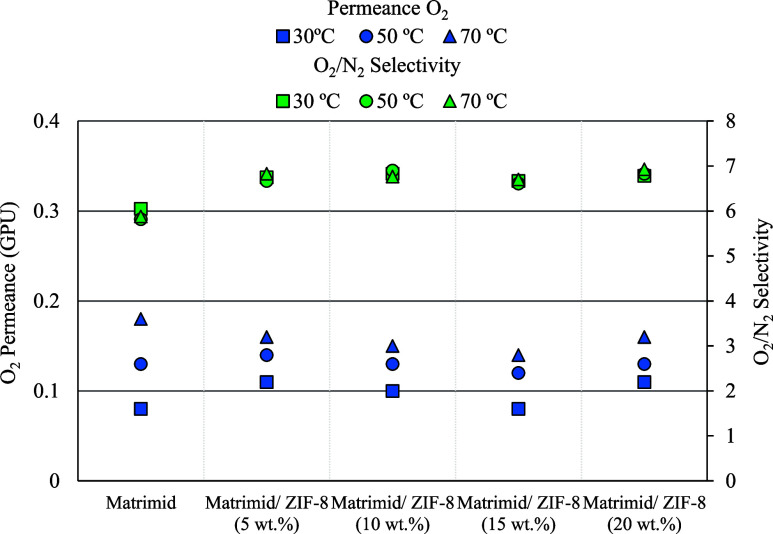
O_2_ permeance and O_2_/N_2_ selectivity of the flat-sheet membranes (feed gas mixture: oxygen/nitrogen 50:50 mol ratio).

Regarding permeability, there is a clear trend in all the membranes studied: it increases with the rise in temperature in the range of 30 to 70 °C. This is due, in part, to the increased mobility of polymer chains with temperature. Concerning the inclusion of ZIF-8 in the polymeric matrix, the results show that the addition of this filler (5 wt %) significantly enhances the membrane permeability. This is in agreement with studies reported in the literature, where the addition of ZIF-8 in a Matrimid matrix improved its permeability.^29,40^ Furthermore, it has been observed that a further increase in ZIF-8 concentration up to 20 wt % does not provide a substantial improvement in membrane permeability, leading to the conclusion that 5 wt % is sufficient to observe an improvement in permeability. On the other hand, the inclusion of ZIF-8 has improved the O_2_/N_2_ selectivity by approximately one point and exhibited a similar behavior in terms of permeability: with a 5 wt % loading of ZIF-8, a considerable improvement is observed without increasing selectivity as more fillers are added. One of the factors that explains this behavior is that the internal structure and surface morphology remain the same over the range of ZIF-8 concentrations in Matrimid studied (5 wt %–20 wt %). Figure S2 shows the cross section of a Matrimid membrane with 20 wt % of ZIF-8. As can be seen, the internal structure is similar to that shown in Figure 3d, where it is seen with 5 wt % in ZIF-8. This increase in O_2_/N_2_ selectivity due to ZIF-8 has been observed in previous works in the literature for other types of polymers like poly­(ether sulfone) and PIM-1,^26,41^ which is consistent with the increase in oxygen selectivity seen in this work. In this way, other authors have found similar behavior in mixed matrix polymeric membranes when including ZIF-8 as a filler. Azam et al. developed cellulose acetate flat-sheet membranes incorporating ZIF-8 into the polymer matrix and tested them for oxygen and nitrogen separation. They found that a 5 wt % loading of ZIF-8 was optimal for enhancing membrane performance and that beyond this composition the membrane actually lost selectivity.^35^

#### 2.2.2 Hollow Fiber Membrane Performance

The evaluation of hollow fiber membranes has been conducted similarly to flat-sheet membranes but with slight variations. In this case, it is not feasible to accurately determine the thickness of the selective dense layer present in the outer surface of the membrane, so it is not possible to calculate its permeability, only the permeance to O_2_ and N_2_, as well as the O_2_/N_2_ selectivity. The temperature range studied is also between 30 and 70 °C, while the partial pressures studied for both compounds go up to 2.5 bar. As with the flat-sheet membranes, using eq 2 which relates the flux of each component *i* to its partial pressure gradient, membrane permeance values have been obtained. As an example, Figure S3 shows the O_2_ flux versus oxygen partial pressure gradient for the hollow fiber membrane composed only of Matrimid.

Two types of membranes in the hollow fiber configuration have been developed: on one hand, a membrane entirely composed of Matrimid and, on the other hand, one in which ZIF-8 has been added as a filler to the Matrimid. As already mentioned, there are studies in the literature where ZIF-8 is added to flat-sheet polymeric membranes, but there are few studies involving polymeric membranes in a hollow fiber configuration with fillers. Additionally, many authors have worked with hollow fiber membranes and obtained dense selective layers with defects on their outer surface, making it necessary to use a protective layer of a rubbery polymer. The novelty of this work lies in the development of Matrimid hollow fiber membranes with a defect-free dense selective outer layer and the addition of ZIF-8 to this material in a hollow fiber configuration. Figure 6 shows the results in terms of the O_2_ permeance and the O_2_/N_2_ selectivity for these two membranes and for the working temperature range. The feed gas composition was oxygen and nitrogen with a 50:50 mol ratio. The numerical values represented in this figure are provided in Table S2.

**Figure 6:**
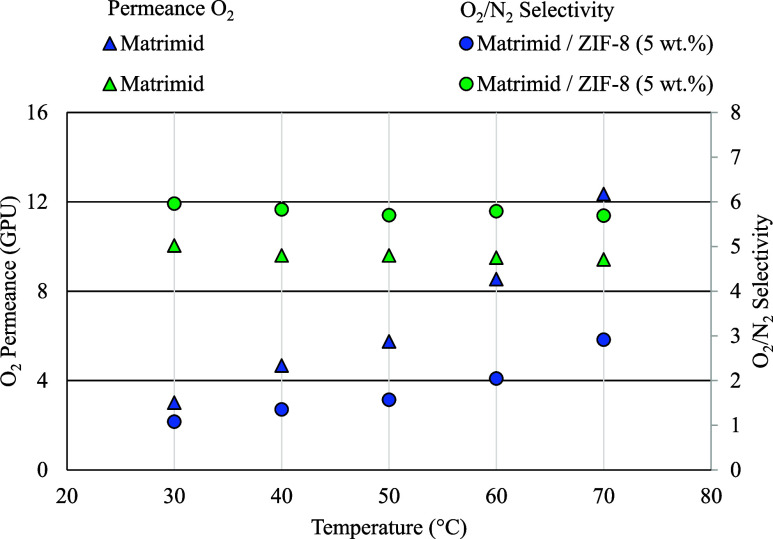
O_2_ permeance and O_2_/N_2_ selectivity of the hollow fiber membranes (feed gas mixture: oxygen/nitrogen 50:50 mol ratio).

As can be seen, the transition from flat-sheet membranes to a hollow fiber configuration allows for a considerable gain in terms of permeance (from 0.13 to 5.75 GPU of O_2_ at 50 °C for Matrimid). This is due to the possibility of hollow fibers to develop much thinner dense selective layers, although this comes at the cost of losing some oxygen selectivity (from 5.82 to 4.80 at 50 °C for Matrimid). The dependence of permeance on temperature for this type of membrane is similar to that of flat-sheet membranes, increasing with temperature. Despite this loss in selectivity, the gain in productivity with the change in membrane configuration is very significant.

As already mentioned in this work, it is not possible to accurately determine the thickness of the selective dense layer in hollow fiber membranes. However, knowing the permeance and since permeability is an intrinsic property of the material, a theoretical thickness for these membranes can be obtained. This is possible because the permeability of Matrimid was first obtained in this work for membranes in a flat-sheet configuration. In this case, for both hollow fiber membranes, the calculated theoretical thickness of the selective dense layer is around 2 μm, which is in accordance with what can be seen in the SEM images (Figure 4b,d), where an external dense layer of few micrometers can be distinguished.

The inclusion of ZIF-8 at 5 wt % in the Matrimid hollow fiber membrane gives an increase of about 1 point in selectivity compared to the pure Matrimid membrane. This is a very significant improvement and is consistent with what happens with flat-sheet membranes, where selectivity also increased when ZIF-8 was included in the polymer matrix. This improvement in selectivity due to ZIF-8 compensates for the loss that occurs when working in a hollow fiber configuration, making the productivity of these membranes very competitive and among the best in the literature in terms of oxygen and nitrogen separation.

### 2.3 Feed Composition Influence

The behavior of hollow fiber membranes has also been studied for different feed compositions and for pure gases (oxygen and nitrogen) at 50 °C. This has been done in order to check whether there is any competitive phenomenon between both molecules when diffusing through the membrane that could hinder or modify the separation at certain composition ranges. Table 1 shows the permeance values for both oxygen and nitrogen as well as the oxygen selectivity in the case of hollow fiber membranes when the feed composition is varied.

**Table 1:** O_2_ and N_2_ Permeance and O_2_/N_2_ Selectivity of the Hollow Fiber Membranes for Different Feed Gas Compositions at 50 °C

Membrane	Feed composition (O_2_ vol %/ N_2_ vol %)	Permeance O_2_ (GPU)	Permeance N_2_ (GPU)	Selectivity O_2_/N_2_
Matrimid 26 wt %	100/0	5.38	-	-
	75/25	5.56	1.12	4.96
	50/50	5.75	1.19	4.80
	25/75	6.14	1.20	5.09
	0/100	-	1.15	-
Matrimid 25 wt %/ZIF-8 (5 wt %)	100/0	2.80	-	-
	75/25	2.95	0.52	5.71
	50/50	3.14	0.55	5.70
	25/75	3.09	0.51	6.09
	0/100	-	0.47	-

As can be seen, there is a slight variation in the permeance values for both oxygen and nitrogen as the feed composition changes. However, this deviation is in any case less than 15%, which can be attributed to the experimental error inherent in carrying out the different experiments. With regard to the oxygen selectivity, this deviation is also minimal in all cases. Therefore, given the consistency of the results obtained, it can be concluded that the variation in the oxygen and nitrogen compositions of the feed does not significantly affect the performance of the membrane in the separation process.

### 2.4 Robeson Plot

In light of the results, it is clear that the addition of ZIF-8 as a filler in Matrimid polymeric membranes significantly improves their performance in terms of oxygen/nitrogen selectivity. Matrimid is a polymer that is already one of the best materials for membrane development and oxygen permeation. Therefore, the possibility of producing hollow fiber membranes from this material and the improvement provided by the inclusion of ZIF-8 in its polymer matrix make the membranes developed in this work some of the most competitive in the literature, which are represented in the plot as hole dots. These data were obtained from the CSIRO (Virtual Screening of Materials) membrane database, making the appropriate changes from permeability to permeance according to each point.^42^ As a proof of this, Figure 7 shows the Robeson plot for oxygen/nitrogen separation. This representation enables different membranes to be compared with respect to their permeance (or permeability) to a gas and their selectivity. This Robeson plot has been elaborated by us based on the data from the above-mentioned database. Moving to the right and up, the membranes perform better, and as can be seen, the squares representing the Matrimid membranes with ZIF-8 are among the best. Some of the data plotted in Figure 7, which show good results, such as those on the right-hand side of the graph, correspond to materials that may be unstable over time, like polymers with intrinsic microporosity (PIMs) or ceramic membranes, which are very expensive to manufacture and maintain. It is also important to note that the values reported in this work have been obtained by continuous permeation with gas mixtures, while many values reported in the literature have been obtained by permeation of pure gases using the time-lag technique, and therefore, the reported selectivity corresponds to the ideal selectivity.

**Figure 7:**
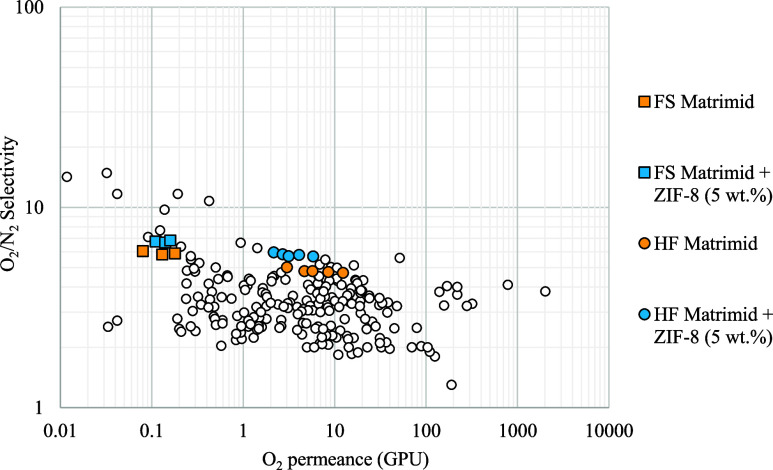
Robeson plot for oxygen selective membranes.

## 3 Discussion and Conclusions

In this work, membrane technology has been applied for the separation of two highly interesting compounds present in air, namely, oxygen and nitrogen. The base material for the development of the membranes was Matrimid, and its effectiveness was studied in both flat-sheet and hollow fiber configurations. Additionally, ZIF-8 was added as a filler in both configurations to enhance the performance. The techniques used in this work for membrane fabrication were casting solvent evaporation (for flat membranes) and spinning (in the case of hollow fiber configuration).

The results have shown, first, how the transition from a flat-sheet membrane configuration to a hollow fiber one has significantly increased the permeance (from 0.13 GPU to 5.75 GPU of O_2_ at 50 °C for Matrimid membranes) at the expense of a slight loss in selectivity (from 5.82 to 4.80 for the same membrane). This is quite reasonable since the hollow fiber configuration allows the development of a very thin dense selective layer, thereby increasing productivity. On the other hand, it was observed that the addition of a filler such as ZIF-8 considerably improved the performance of Matrimid in the separation of oxygen and nitrogen molecules. Specifically, the most significant improvement is the increase in oxygen selectivity by one point. Furthermore, it has been found that a 5% weight loading in the polymer matrix is enough to observe these effects without the need to increase it further.

Due to the close kinetic diameters of oxygen and nitrogen molecules (3.46 and 3.64 Å, respectively), their separation can be quite challenging, making the choice of materials for membrane design critical. ZIF-8 was chosen as a filler to boost the performance of Matrimid in the separation of oxygen and nitrogen for several reasons. First, it is a material with high thermal stability up to 550 °C and chemical resistance. In addition, it has one of the highest surface areas and pore volumes among zeolitic structure fillers, 1752 m^2^ g^–1^ and 0.634 cm^3^ g^–1^, respectively, which increases the specific surface area of the membrane and thus the adsorption area.^43,44^ Furthermore, ZIF-8 has been shown to be effective in separating smaller gas molecules from larger ones through a molecular sieving effect.^29^ The pore size of ZIF-8 is 11.6 Å, but these pores are connected by cavities with an aperture of 3.4 Å, as demonstrated by single-crystal X-ray diffraction. This size is close to the oxygen kinetic diameter although slightly smaller. Nevertheless, Zhang et al. found evidence that these cavity openings in ZIF-8 are somewhat flexible at room temperature (35 °C), which would explain why oxygen molecules can pass through them, while nitrogen molecules are rejected by the molecular sieving effect, as this chain flexibility is not unlimited and would exclude larger molecules.^45^ In the same study, Zhang demonstrated the unexpected molecular sieving behavior of ZIF-8 by estimating the thermodynamically corrected transport diffusivities of various molecules through this material. In the case of oxygen, the corrected diffusivity is 1·10^–5^ cm^2^ s^–1^, while for nitrogen, it is 4·10^–6^ cm^2^ s^–1^. They also found that pure ZIF-8 has a higher permeability to oxygen than to nitrogen. These permeability data were back-calculated using the Maxwell model, employing experimental permeation data for pure gases through the polyimide 6FDA-DAM and with mixed matrix membrane (ZIF-8/6FDA-DAM) dense films. This model is the most widely used to predict the permeability of composite materials. These facts would explain the greater affinity for oxygen over nitrogen observed in our work and the performance improvement seen in the results, particularly in terms of oxygen selectivity, when ZIF-8 is included in the polymer matrix of the developed membranes.

A study has also been conducted on the behavior of Matrimid and Matrimid with ZIF-8 hollow fiber membranes when changing the feed composition. It was found that this variation does not affect the performance of the separation process. On the other hand, it is worth highlighting that the main novelty of this work is the inclusion of ZIF-8 in Matrimid hollow fiber membranes, especially considering the difficulty of incorporating fillers into polymeric membranes in a hollow fiber configuration. The improvement provided by this material places the membranes developed in this work among the best in comparison with those available in the literature.

## 4 Materials and Methods

### 4.1 Materials

Matrimid 5218 polyimide (CAS n° 104983-64-4) was employed as the primary polymer for this study and was kindly supplied by Huntsman (USA). Dichloromethane (CAS n° 75-09-2) and 1-methyl-2-pyrrolidone (NMP) (CAS n° 872-50-4) were used as Matrimid solvents for membrane preparation, while methanol (CAS n° 67-56-1) was used in the drying step of the membranes. Dichloromethane was employed in the case of flat-sheet membranes and NMP for hollow fiber membrane production. Dichloromethane was provided by Honeywell, whereas methanol and NMP were purchased from Sigma-Aldrich (Emplura). ZIF-8 was purchased from ACSYNAM, and its chemical structure can be seen in Figure 8.

**Figure 8:**
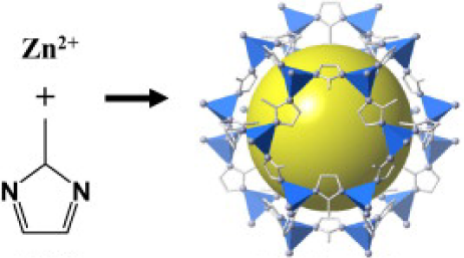
Crystal structure of ZIF-8: Zn (polyhedral), N (sphere), and C (line). Adapted from Lee et al.^46^ with permission.

The gases used in this work, oxygen (purity >99.995%), nitrogen (purity ≥99.999%), and helium (purity ≥99.999%), were purchased from Nippon Gases, Spain, S.L.U.

### 4.2 Flat-Sheet Membranes

Flat-sheet membranes were prepared by using the casting solvent evaporation technique (Figure 9). Five types of membranes were prepared, including pristine Matrimid membranes and membranes with different ZIF-8 loadings in the Matrimid matrix (5, 10, 15, and 20 wt %). First, 0.3 g of Matrimid were weighed and dissolved in 5 mL of dichloromethane solvent under continuous stirring for 1–2 h. The resultant solution was then poured into a Petri dish to form a membrane-like shape and left at room temperature to allow solvent evaporation for 1 day. Upon complete solvent evaporation, the membrane was peeled off from the Petri dish, and its thickness was measured using a digital micrometer. In the case of membranes with ZIF-8, the process is similar to some variations when preparing the solution. On one hand, half of the 5 mL of dichloromethane is used to dissolve Matrimid, while the other half is used to disperse the ZIF-8 particles. The ZIF-8 dispersion is subjected to three stirring and ultrasonic cycles of 20 min each. Finally, it is mixed with the Matrimid solution and undergoes three additional 20 min cycles of stirring and ultrasound.

**Figure 9:**
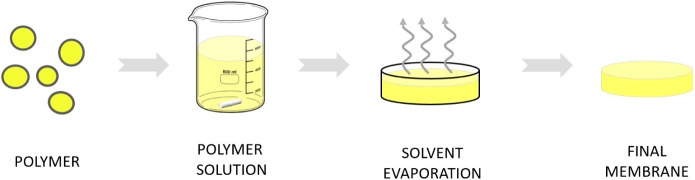
Flat-sheet membrane production scheme.

### 4.3 Spinning of Hollow Fibers

The dry-wet spinning method is employed for the fabrication of hollow fiber membranes. The key component of this process lies in the spinneret, which gives the polymer a hollow fiber shape when it is extruded and then coagulates in the coagulation bath. In this work, Matrimid hollow fiber membranes were successfully produced without defects on their surface layer by using this method. The coagulation tank and the entire pulley system are homemade, while the spinneret was purchased from EMI Twente. The schematic diagram of the process can be found in a previous work.^9^

In this work, two types of hollow fiber membranes have been developed, both with Matrimid as the base material. First, a hollow fiber membrane composed only of Matrimid has been manufactured, while, on the other hand, ZIF-8 has been included in the polymeric matrix, resulting in a mixed matrix hollow fiber membrane composed of Matrimid and ZIF-8.

In the first case, the spin dope used for the spinning process is composed of 26 wt % Matrimid and 74 wt % NMP. To make it, the NMP is heated up to 50 °C and placed under mechanical stirring. The entire amount of Matrimid is added to the NMP gradually, taking care not to form agglomerates, and once it has all been added, it is left stirring for 6 h. It is important to always maintain a constant temperature of about 50 °C. When it is completely dissolved, the agitation is stopped, and it is left to degas for 24 h in an oven, maintaining the established temperature. The concentration ratio used is necessary so that microdefects are not formed in the dense surface of the Matrimid membrane during the spinning process.^47^ When the solution is ready to be extruded, both the spin dope and the bore liquid are poured into two stainless steel syringes. The bore liquid used is a 20 wt % aqueous solution of NMP, prepared with ultrapure water. This is done with the intention of creating a highly porous zone with large macro voids in the lumen side of the hollow fiber membranes.^48,49^ In contrast, a dense selective layer is designed to form on the outer surface of the membrane, so the coagulation bath that contacts this part is water. When the hollow fiber membranes are finished being extruded, they are left to coagulate in water for 72 h to remove any remaining solvent and finish coagulating. Finally, to dry them, three 20 min methanol baths are applied, and then, they are left to dry at room temperature.

In the case of mixed matrix hollow fiber membranes, the process to follow is similar. The spin dope used consists of 25 wt % of the Matrimid/ZIF-8 mixture, where the filler makes up 5% of that amount. The remaining 75 wt % corresponds to the solvent (NMP). The operating conditions used in the spinning process for this membrane are the same as in the first case, with the aim of maintaining a similar membrane structure. The parameters and operating conditions used for spinning in both cases can be found in Table 2.

**Table 2:** Parameters Used in the Matrimid Spinning Process

Polymer content in spin dope (wt %)	26	25
Filler load (wt %)	0	5
Solvent	NMP	
Dope flow rate (mL min^–1^)	3	
Dope extrusion temperature (°C)	50	
Bore liquid	NMP (20 wt %)/ ultrapure water(80 wt %)	
Bore liquid temperature (°C)	20	
Bore liquid flow rate (mL min^–1^)	1.5	
External coagulant	Water	
External coagulant temperature (°C)	20	
Air gap (cm)	16	
Coagulation bath depth (m)	1.5	

### 4.4 Membrane Characterization

In the case of flat-sheet membranes, a micrometer (Mitutoyo model 293-821) was used to determine their thickness. In addition, for the morphological characterization of both flat-sheet and hollow fiber membranes, scanning electron microscopy (SEM) was used. This technique was carried out in the laboratory of the Materials Science and Engineering Division (LADICIM), at the University of Cantabria, with a Zeiss EVO MA15 microscope. All samples were frozen with liquid nitrogen to obtain a cross-sectional view by brittle fracture. The goal of using this technique was to avoid deforming the polymer and to obtain high precision when the samples were observed under the microscope. Furthermore, for the chemical characterization and elemental analysis of the material present in the membrane, elemental maps were performed by energy disperse X-ray analysis (EDX) using a 10 mm^2^ silicon drift detector from Oxford Ins (model X-act), operated by INCA software. This was done in order to verify the presence of ZIF-8 in the corresponding membranes. The data generated by this analysis provided a spatial distribution of zinc through a mapping.

### 4.5 Gas Permeation Experiments

The gas permeation tests of the membranes were carried out in a laboratory-scale experimental plant, as shown in Figure 10. In the case of flat-sheet membranes, a 9 cm diameter stainless steel cell was used with openings for all the gas streams of the process (feed, retentate, sweep gas, and permeate). The effective area available in this case is 50.2 cm^2^. On the other hand, for testing of hollow fiber membranes, stainless steel modules were built and 5 fibers were introduced into each one. The useful length of these fibers was 15 cm, and they were effectively sealed at the edges of the modules using epoxy resin, so the effective area is about 20 cm^2^. For both membrane modules, a mixture of oxygen and nitrogen with a 50:50 mol ratio was fed, and helium was used as sweep gas in order to maintain the maximum partial pressure gradient across the membrane. In addition, the performance of hollow fiber membranes was also studied for different feed compositions and for pure gases. In the case of hollow fiber membranes, the feed was introduced by the shell side and the sweep gas through the lumen, from which the permeate was also collected.

**Figure 10:**
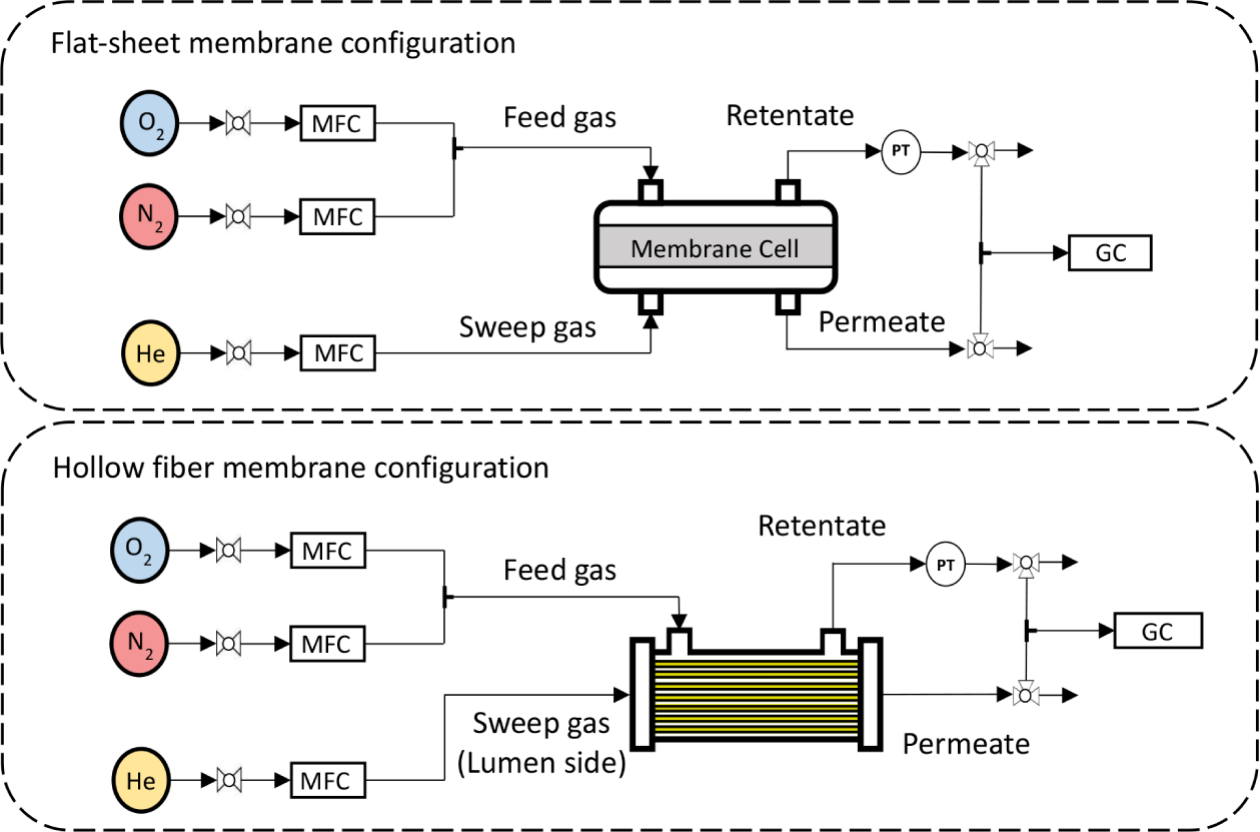
Schematic diagram of the experimental setup. MFC, mass flow controller. PT, pressure transducer. GC, gas chromatograph.

The performance of each membrane was assessed in terms of permeability, permeance, and oxygen-to-nitrogen selectivity. To obtain these parameters, it is necessary to obtain first the permeation flux across the membrane of each compound (*J*_*i*_), which is calculated by the ratio of the amount of permeate per unit time to the total membrane area as follows:

1 $$J_{i}=\frac{Q_{i}}{A_{m}},\left[Q_{i}\right]=cm^{3}\left(STP\right)\;s^{-1}$$

where *Q*_*i*_ is the flow of each compound permeating across the membrane and *A*_m_ is the membrane area. In the case of hollow fiber membranes, the diameter of the outer part of the membrane (which corresponds to the dense selective layer) was used to obtain the total area. In gas separation processes using these types of membranes, the transport of gases through them is described by the solution-diffusion mechanism, where the driving force is the pressure gradient across the membrane.^50^ Then, with the flux of each component through the membrane, permeance (*P*_*i*_) can be obtained, which is given by

2 $$P_{i}=\frac{J_{i}}{\Delta p_{i}}$$

where Δ*p*_*i*_ is the partial pressure gradient across the membrane, and for a hollow fiber membrane, module working in cocurrent is obtained with the following equation:

3 $$\Delta p_{i}=\frac{\left(p_{i,feed}-p_{i,permeate\left(0\right)}\right)-\left(p_{i,retentate}-p_{i,permeate}\right)}{\ln\left(\frac{p_{i,feed}-p_{i,permeate\left(0\right)}}{p_{i,retentate}-p_{i,permeate}}\right)}$$

where *p*_*i,* feed_ is the partial pressure of component *i* in the feed, *p*_*i,*permeate(0)_ is the partial pressure of component *i* in the permeate at an initial position, *p*_*i,*retentate_ is the partial pressure of component *i* in the retentate stream, and *p*_*i,*permeate_ is the partial pressure of component *i* in the permeate stream.

To determine the membrane permeability to each compound (permeability_*i*_), it is essential to know the thickness of the selective layer (δ) of the membrane. In the case of flat-sheet membranes, this is relatively straightforward, as the entire membrane body constitutes this layer (these membranes have an isotropic structure). However, for hollow fiber membranes (with an asymmetrical structure), this task becomes challenging because SEM images do not allow the thickness of the selective layer to be accurately assessed. It is for this reason that the calculation of permeability is carried out only for membranes in a flat-sheet configuration. It is calculated as

4 $$Permeability_{i}=P_{i}\cdot \delta$$

Lastly, the selectivity of the membrane toward a compound (α_*i*/*j*_) is calculated as the ratio between the permeance (or permeability) of one compound to another.

5 $$\alpha_{i/j}=\frac{P_{i}}{P_{j}}$$

As for the work in the laboratory, the experiments were conducted within the temperature range of 30 to 70 °C. This span allows for exploration of membrane behavior across varying thermal conditions, ensuring robust performance assessments. The variation of partial pressures for individual compounds ranged from 0.5 to 5 bar. To enhance the result’s reliability, five samples were collected under each operating condition, minimizing experimental error and providing statistically significant data for subsequent analysis. For sample analysis, an Agilent chromatograph model “8860 GC system” with a single-filament thermal conductivity detector (TCD) was used, which allows for precise quantification of the concentration of each compound in the gaseous streams.

## Supplementary Material



## Notes

Relationship between flux and pressure gradient; oxygen permeation flux as a function of oxygen partial pressure gradient for the flat-sheet Matrimid membrane (Figure S1) and for the pristine Matrimid hollow fiber membrane (Figure S3); the SEM image of the cross-sectional view of a flat sheet Matrimid membrane with 20 wt % of ZIF-8 (Figure S2); O_2_ permeability, O_2_ permeance, and O_2_/N_2_ selectivity values of the flat-sheet membranes (Table S1) and of the hollow fiber membranes (Table S2) (PDF)
