# Supplementary material for: Matrimid Mixed Matrix Hollow Fiber Membranes: Influence of ZIF‑8 Filler over O2/N2 Separation Performance
Source: ACS Omega. 2025 May 29;10(22):22813–24. doi: 10.1021/acsomega.4c11696 (PMC12163829; doi:10.1021/acsomega.4c11696)
Supplement: Supplementary file 1 [file ao4c11696_si_001.pdf]

Supporting Information

Matrimid mixed matrix hollow fiber membranes: Influence of ZIF-8 filler over O_2/N_2 separation performance

Authors: Daniel González-Revuelta[(1)], Marcos Fallanza[(1)], Alfredo Ortiz[(1)], Daniel Gorri[(1)] *

[(1)] Departamento de Ingenierías Química y Biomolecular, Universidad de Cantabria, Av. de los Castros s/n, 39005 Santander, Spain

* email: gorrie@unican.es.

Relationship between permeation flux and pressure gradient:

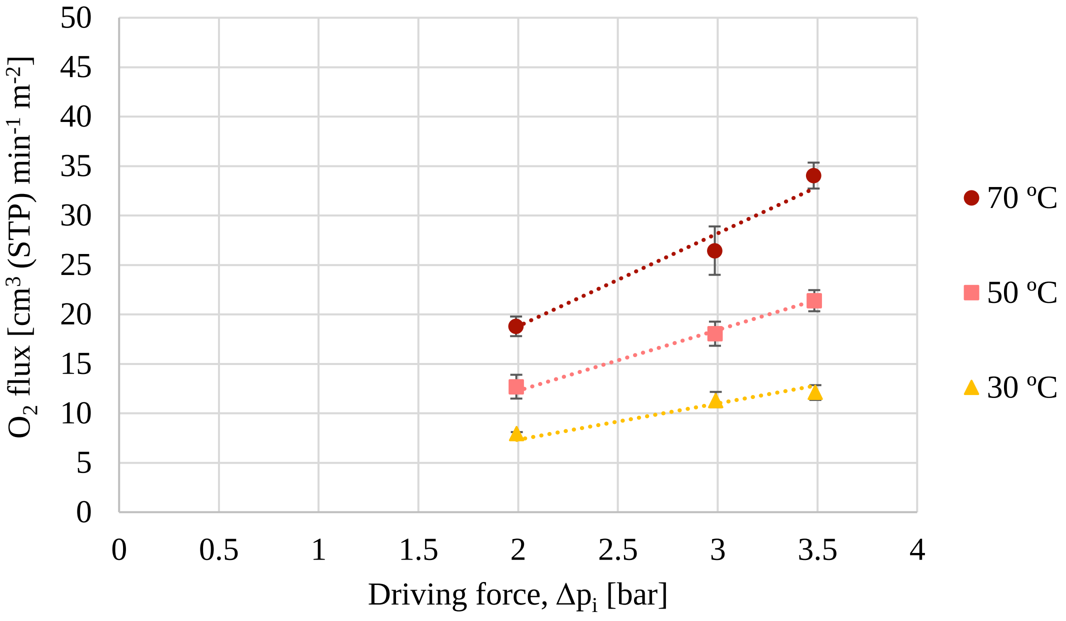

Figure S1. Oxygen permeation flux as a function of oxygen partial pressure gradient for flat-sheet Matrimid membrane. Feed mixture: binary 50:50 oxygen/nitrogen mixture.

Mixed Matrix membrane SEM image:

Figure S2 shows a cross section view of a Matrimid flat sheet membrane with a 20 wt.% of ZIF-8.

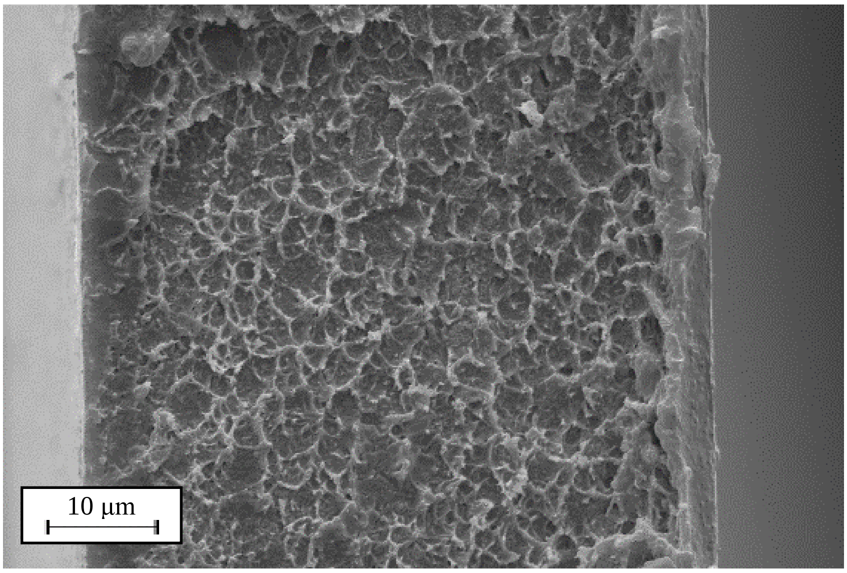

Figure S2. SEM image: cross section of Matrimid + 20 wt.% ZIF-8 membrane.

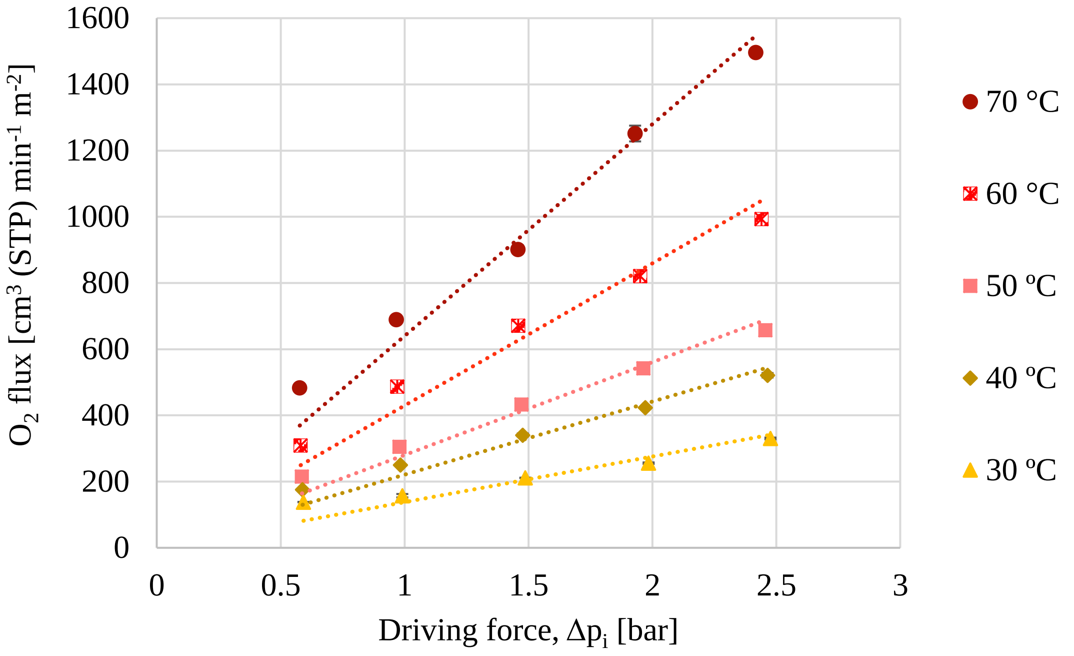

Figure S3. Oxygen permeation flux as a function of oxygen partial pressure gradient for pristine Matrimid hollow fiber membrane. Feed mixture: binary 50:50 oxygen/nitrogen mixture.

Permeability, permeance, selectivity and thickness values:

Table S1 shows the O_2 permeance, O_2 permeability, and O_2/N_2 selectivity values for each of the membranes for different temperatures when working with a gas mixture feed of oxygen and nitrogen with a 50:50 mol ratio. Additionally, the thicknesses of each of them are also shown.

Table S1. O_2 permeability, O_2 permeance and O_2/N_2 selectivity values of the flat-sheet membranes. (Feed gas mixture: oxygen/nitrogen 50:50 mol ratio).

Membrane	T (°C)	Permeability O_2 (Barrer)	Permeance O_2 (GPU)	Selectivity O_2/N_2	Thickness (μm)
Matrimid	30	2.6	0.08	6.05	32
	50	4.0	0.13	5.82	
	70	5.8	0.18	5.88	
Matrimid/ ZIF-8 (5 wt.%)	30	4.9	0.11	6.75	42
	50	5.9	0.14	6.67	
	70	7.0	0.16	6.83	
Matrimid/ ZIF-8 (10 wt.%)	30	4.8	0.10	6.83	50
	50	6.3	0.13	6.90	
	70	7.3	0.15	6.77	
Matrimid/ ZIF-8 (15 wt.%)	30	4.2	0.08	6.67	55
	50	6.8	0.12	6.61	
	70	7.9	0.14	6.70	
Matrimid/ ZIF-8 (20 wt.%)	30	5.6	0.11	6.78	50
	50	6.7	0.13	6.83	
	70	7.8	0.16	6.93	

Table S2 shows the O_2 permeance and O_2/N_2 selectivity values for the two hollow fiber membranes in the working temperature range. The feed gas composition was oxygen and nitrogen with a 50:50 mol ratio.

Table S2. O_2 permeance and O_2/N_2 selectivity values of the hollow fiber membranes. (Feed gas mixture: oxygen/nitrogen 50:50 mol ratio).

Membrane	T(°C)	Permeance O_2(GPU)	Selectivity O_2/N_2
Matrimid 26 wt.%	30	3.01	5.02
	40	4.67	4.80
	50	5.75	4.80
	60	8.54	4.75
	70	12.35	4.71
Matrimid 25 wt.% / ZIF-8 (5 wt.%)	30	2.16	5.96
	40	2.71	5.83
	50	3.14	5.70
	60	4.09	5.79
	70	5.83	5.69
